# Drug-Resistant *Mycobacterium tuberculosis* Isolates from New and Previously Treated TB Patients in China, 2017-2019

**DOI:** 10.1590/0037-8682-0728-2020

**Published:** 2021-03-22

**Authors:** Zeng Mei Chun, Jia Qing Jun

**Affiliations:** 1 Zhejiang University, College of Medicine, The First Affiliated Hospital, Department of Pathology, Hangzhou, Zhejiang, China.; 2 Hangzhou Center for Disease Control and Prevention, Department of TB Control and Prevention, Hangzhou, Zhejiang, China.

**Keywords:** M. tuberculosis, Drug resistance, Gene mutations, Gene sequencing

## Abstract

**INTRODUCTION:**

*Mycobacterium tuberculosis* (MTB) is a causative agent of tuberculosis (TB) that causes death worldwide.

**METHODS:**

MTB was subjected to phenotypic drug-susceptibility tests (DST), and drug-resistant genes were sequenced.

**RESULTS:**

Previously treated patients were more likely to have positive smear results and exhibit drug resistance. New patients were more likely to be mono SM-resistant and less likely to be INH- and RIF-resistant. The most common mutations were *katG* (S315T), *rpoB* (S450L), *rpsL* (K43R), and *embB* (M306V).

**CONCLUSIONS:**

The proportion of mono-SM-resistant TB among new patients was higher.

Tuberculosis (TB), usually caused by *Mycobacterium tuberculosis* (MTB), remains a major threat to the public health worldwide. Although the directly observed treatment, short-course (DOTS) strategy has significantly reduced the incidence of TB in recent years, the emergence of drug-resistant TB has severely hampered TB prevention and control, especially regarding multidrug-resistant TB (MDR-TB) and extensively drug-resistant TB (XDR-TB)^1^. 

TB epidemics are unevenly distributed in China, and there is a high prevalence in rural areas, especially those that are underdeveloped in the northwest and southwest of China^1^. Migration, primarily from rural to urban environments, has become common in China in recent decades. Internal migration presents a significant challenge to the national TB control strategies. The latest national survey revealed that the frequency of MDR-TB among pulmonary TB patients in China was 8.32%^1^. MDR-TB is a serious clinical and epidemiological problem, incurring substantial economic management costs, as treating patients resistant to isoniazid (INH) and rifampicin (RIF) may be many times more expensive than treating those for whom the main medications are effective. Many studies have investigated the mechanisms of resistance to INH, RIF, streptomycin (SM), and ethambutol (EMB). Mutations in the *katG* gene are a major cause of INH resistance^2^. MTB can acquire resistance to RIF through mutations in the *rpoB* gene, especially in the 81-bp RIF resistance determining region (RRDR)^3^. Mutation-carrying genes, such as *rpsL* and *rrs*, which encode the S12 ribosomal protein and 16S rRNA, respectively, are associated with intermediate or high levels of SM resistance^4^. Point mutations in *embB* codon 306, which occur in 30%-69% of clinical EMB-resistant strains, are associated with resistance to EMB^5^. Thus, the identification of mutations, especially in *katG, inhA, rpoB, rpsL, rrs*, and *embB*, is thought to represent a rapid screening method for the detection of first-line drug resistance in clinical isolates.

The current analysis presents data on the drug resistance profiles of drug-resistant TB as well as gene mutations from a larger MTB DNA sample of the most recent prevalent drug-resistant isolates in rural areas of China. Based on positive sputum culture specimens, MTB isolates were identified, phenotypic drug sensitivity tests (DST) were conducted, and drug-resistant genes were sequenced. 

Patient information was registered and verified at the Community Health Care Center. Specimens were collected from specialist TB hospitals in Hangzhou, China, from 2017 to 2019. Sputum samples were collected from patients suspected to have TB. Samples were subjected to acid-fast staining and microscopy tests and were cultured on Lowenstein-Jensen medium according to the national guidelines^6^ (Figure 1 in supplemental file). 

Samples were submitted to the tuberculosis reference laboratory of the Hangzhou Center for Disease Control and Prevention. Phenotypic DSTs for each drug were determined using the proportional method on Lowenstein-Jensen medium. The concentrations of INH (0.2 mg/L), RIF (40 mg/L), SM (4.0 mg/L), and EMB (2.0 mg/L. Sterile deionized water (ddH_2_O) and a standard strain of H37Rv (American Type Culture Collection 27294) were used as negative and positive controls, respectively, in all experiments. Drug-resistant MTB genomic DNA was extracted and stored at 20 °C for further use. Genetic fragments associated with drug resistance were amplified using previously reported primers^7^. Purified PCR products were sent to Sangon Biotech (Shanghai, China) for sequencing. Sequences were aligned with the published sequences (GenBank accession number NC_000962). 

Categorical data were summarized as counts or percentages (%), and the groups were compared using chi-square test or Fisher's exact test. Statistical significance was set at *p* < 0.05. SPSS 11.0 (IBM SPSS Statistics, United States) software was used for the statistical analyses.

As shown in Table 1, among 2 578 TB patients, the number of male patients was higher than that of female patients in each group (χ^2^ = 23.861, *p* < 0.001). There were more patients between 76 and 91 years old in the new group (47.80%) than in the previously treated group (27.60%; χ^2^ = 91.690, *p* < 0.001). The group with no TB treatment history comprised more of clerks (χ^2^ = 48.625, *p* < 0.001), retirees (χ^2^ = 109.309, *p* < 0.001), and residents (χ^2^ = 81.078, *p* < 0.001). Previously treated patients were more likely than new patients to have positive smear results (χ^2^ = 41.079, *p* < 0.001) and drug resistance (χ^2^ = 196.419, *p* < 0.001).

**TABLE 1:** Characteristics of 2578 TB patients in Hangzhou.

Characteristic	Treatment history (N=2 578)		***p* value** ^§^
	New patients	Previously treated patients	
	(n=1 933, %)	(n=645, %)	
Gender			
Male	1 178 (60.94)	462 (71.63)	Reference
Female	755 (39.06)	183 (28.37)	<0.001
Age, years			
<45	323 (16.71)	113 (17.52)	0,002
46~75	686 (35.49)	354 (54.88)	Reference
>76	924 (47.80)	178 (27.60)	<0.001
Occupation			
Worker	526 (27.21)	239 (37.05)	Reference
Farmer	678 (35.08)	304 (47.13)	0,899
Clerk	211 (10.92)	19 (2.95)	<0.001
Retired	394 (20.38)	20 (3.10)	<0.001
Others	124 (6.41)	63 (9.77)	0,519
Census			
Resident	1 571 (81.27)	413 (64.03)	Reference
Migrant	362 (18.73)	232 (35.97)	<0.001
Treatment outcome			
On treatment	267 (13.81)	89 (13.80)	Reference
Cure	714 (36.94)	278 (43.10)	0,271
Complete	835 (43.20)	247 (38.29)	0,401
Failure	102 (5.28)	13 (2.02)	0,002
Others	15 (0.78)	18 (2.79)	<0.001
Laboratory test			
Smear positive	1 012 (52.35)	431 (66.82)	<0.001
GeneXpert MTB positive	62 (3.21)	33 (5.12)	0,026
Drug susceptible	1 665 (86.14)	391(60.62)	<0.001

^§^ *P-values* are for comparisons between new patients and previously treated patients.

Of these, 163 were resistant only to INH, 26 were resistant only to RIF, 168 were resistant only to SM, and 23 were resistant only to EMB (Table 2). Among the 500 drug resistant isolates, 3.45% of isolates (89/2 578) were MDR, 64 were resistant to two drugs, 46 were resistant to three drugs, and 10 were resistant to all drugs. 

**TABLE 2:** Drug resistance profiles of 500 drug resistant TB patients.

Drug resistant profiles^$^		New patients	Previously treated patients	***p* value** ^§^
		(n=255)	(n=245)	
Mono-drug resistant	I^R^-only	74	89	Reference
	R^R^-only	10	16	0,509
	S^R^-only	119	49	<0.001
	E^R^-only	12	11	0,542
				
Poly-drug resistant	I^R^+S^R^	13	6	Reference
	I^R^+E^R^	2	0	0,347
	R^R^+S^R^	1	1	0,599
	I^R^+S^R^+E^R^	3	5	0,135
				
Multi-drug resistant	I^R^+R^R^+S^R^	10	16	Reference
	I^R^+R^R^+E^R^	3	9	0,416
	I^R^+R^R^	6	35	0,026
	I^R^+R^R^+S^R^+E^R^	2	8	0,293

^$^ **I:** isoniazid; **R:** rifampicin; **S:** streptomycin; **E:** ethambutol; superscripted "**R":** drug resistant. ^§^ *P-values* are for chi-square or Fisher tests.

Sequencing results of *katG, inhA, rpoB, rpsL, rrs1*, and *embB* genes showed that 68.68% (193/281) carried a single mutation in the *katG* gene at codon 315 (Table 3), 9.25% (26/281) carried a single mutation in the *inhA* gene at codon -15 or -8, and 16.37% (46/281) had mutations in both the *katG* and *inhA* genes. The most frequent mutation was S315T (81.14%, 228/281). For RIF-resistant isolates, 90.60% (106/117) carried mutations in the RRDR of *rpoB*. Seven mutation sites were identified, and the most frequent mutation was at codon 450 (42.74%, 50/117). The second most frequent mutation was found at codon 445 (31.62%, 37/117). In addition, 11.97% (14/117) had mutations at codon 435, while three isolates had synonymous mutations at codons 427, 428 and 430, respectively. Furthermore, 87.55% (204/233) of the SM-resistant isolates harbored mutations in *rpsL* or *rrs1*. The most frequent mutation in the *rpsL* gene was K43R (74.68%, 174/233). A total of 70.91% (39/55) of the EMB-resistant isolates had mutations in the *embB* gene.

**TABLE 3:** Genetic mutations associated with drug resistance.

Drug (Number of isolates)	Locus	Codon/nucleotide changes	Amino acid/ nucleotide changes	Number of isolates (%)
INH (281)	N^#^	N	N	16(5.69)
	*katG*	AGC→ACC	S315T	182 (64.77)
		AGC→AAC	S315N	11(3.91)
	*inhA*	C→T	C(-15)T	17 (6.05)
		T→C	T(-8)C	9(3.20)
	*katG+inhA*	C→T/AGC→ACC	C(-15)T/S315T	27(9.61)
		T→C/AGC→ACC	T(-8)C/S315T	19(6.76)
	N	N	N	11 (9.40)
		ACC→ACG	*T427T* ^*§*^	1 (0.85)
		AGC→TCG	*S428S*	1 (0.85)
		CTG→CTC	*L430L*	1 (0.85)
		CTG→CCG	L430P	1 (0.85)
		GAC→GAT	D435V	1 (0.85)
		GAC→GTC	D435V	13(11.11)
		TCG→CAG	S441Q	1 (0.85)
		TCG→TTG	S441L	1 (0.85)
RIF(117)	*rpoB*	CAC→CGC	H445R	8(6.84)
		CAC→CTC	H445L	14 (11.97)
		CAC→GAC	H445D	6 (5.13)
		CAC→TAC	H445Y	5 (4.27)
		CAC→TGC	H445C	1 (0.85)
		TCG→TTG	S450L	46(39.32)
		TCG→TTT	S450F	2 (1.71)
		TCG→TGG	S450W	1 (0.85)
		CAA→AAA/CAC→CCC	Q432K/H445P	1 (0.85)
		CAC→AAC/TCG→CCG	H445N/S450P	1 (0.85)
		CAC→AAC/CTG →CCG	H445N/L452P	1 (0.85)
SM(233)	N	N	N	29(12.45)
	*rpsL*	AAG→AGG	K43R	174 (74.68)
		AAG→AGG	K88R	7(3.00)
		AAG→AGC	K88S	1(0.43)
		AAG→ACC	K88T	1(0.43)
	*rrs*_*1*_ *(nt 388-1084)*	A→C	A514C	8 (3.43)
		C→T	C517T	13 (5.58)
EMB(55)	N	N	N	16 (29.09)
	*embB*	ATG→ATA	M306I	12 (21.82)
		ATG→GTG	M306V	27 (49.09)

**N** ^#^ **:** No mutation is found; ^§^Synonymous mutations are in italics.

This study analyzed the epidemiology of drug-resistant TB in seven rural areas in Hangzhou (Yuhang, Fuyang, Linan, Tonglu, Jiande, Chunan, and Xiaoshan). Of all included patients, 76.96% (1 984/2578) were Hangzhou residents, and the proportion of previously treated patients was 25.02% (645/2 578), which was significantly lower than the data from other TB hospitals in China^8^. The results indicated a functioning local TB control program in Hangzhou, China. The majority of previously treated patients were male, had positive sputum smear results, and were resistant to RIF (all *P* < 0.05). A total of 20.25% (522/2 578) isolates were resistant to any of the four first-line anti-TB drugs, and the proportion of MDR-TB was 3.45%, much lower than that reported by Lv et al. (31.1% and 10.1%, respectively)^9^. In this study, all TB isolates were collected from specialist TB hospitals in rural areas. We could not obtain all drug-resistant isolates because some patients may seek more highly specialist TB hospitals in urban areas. However, our results revealed, to some extent, the prevalence of drug-resistant TB in the rural areas of Hangzhou. 

Drug-resistant TB is often caused by mutations in genes, especially in *katG* and *inhA* genes for INH resistance, *rpoB* for RIF resistance, *rpsL* and *rrs1* for SM resistance, and *embB* for EMB resistance. Therefore, we analyzed these genes in the current study. The predominance of the S315T substitution in the *katG* gene in INH resistance has been demonstrated globally, and estimates of this mutation range from < 25% to > 90%, and *inhA* also confers low-level INH resistance (> 10%)^2^. We demonstrated that the frequency of the S315T substitution in the *katG* (81.14%) and *inhA* (25.62%) genes was higher than that found by Zhao et al., who reported findings (45.3% and 23.3%, respectively) from the neighboring province of Fujian^7^. The frequency of the S315T mutation was also higher than that found in studies in Jiangxi Province (67.00%)^10^. Resistance to RIF is a well-known surrogate marker of MDR-TB, and mutations in the *rpoB* RRDR remain important RIF resistance markers. In our study, all mutations conferring RIF resistance had mutations within the RRDR, with the most prevalent being S450L (39.32%, 46/117), followed by H445L (11.97%, 14/117). Mutations at codon 445 were the most diverse (H→R/L/D/Y/C/P/N). In agreement with the studies reported previously^10^, 90.60% (106/117) of RIF-resistant isolates had mutations in the RRDR of the *rpoB* gene, and although they were at different locations, most of them were located at three *rpoB* codons: 450, 445, and 435. 

I*nhA* and *katG* genes were the most clinically relevant and determined resistance in most clinical isolates, and this was the main reason we included only *inhA* and *katG* in our study. We found that 5.69% of INH-resistant isolates were not associated with any genotypic mutations in *inhA* or *katG*, which was a much lower result than that reported by Hazbon et al. previously^11^. Furthermore, we reported the frequency of the S315N mutation to be 3.91%, which was much lower than that reported in Taiwan^12^. To date, the S315N mutation has rarely been reported in Hangzhou, China. 

Regarding the two other first-line anti-TB drugs, 87.55% (204/233) of SM-resistant isolates and 70.91% (39/55) of EMB-resistant clinical isolates were identified using molecular techniques. The findings were similar to those reported by Zhao et al. (82.9% and 70.6%, respectively)^7^. Our results showed that 70.91% of EMB-resistant isolates carried mutations at codon 306, which was higher than that reported previously (58.00%)^13^. Of the SM-resistant isolates, 12.45% had mutations in neither *rpsL* nor *rrs1*, and 29.09% of EMB-resistant isolates had no mutations in the *embB* gene, so other related genes may be involved^14^. *GidB* mutations have been found in both resistant and susceptible clinical drug-resistant MTB isolates^15^; therefore, we did not include the *gidB* gene in the analysis we used to detect SM resistance. We sequenced the associated gene fragments of 30 all-drug susceptible MTB isolates simultaneously; however, we found none of the mutations mentioned above.

Our study showed that the overall prevalence of the first-line drug-resistant TB in the rural areas of Hangzhou, China was low. However, the proportion of INH and SM resistance were higher. The most prevalent genetic mutations associated with INH, RIF, SM, and EMB resistance were *katG* (S315T, 81.14%), *rpoB* (S450L, 39.32%), *rpsL* (K43R, 74.68%), and *embB* (M306V, 49.09%), respectively. Additionally, we identified a rare substitution mutation of S450P in the RRDR of the *rpoB* gene. Furthermore, we found that new TB patients were more likely to be resistant only to SM and less likely to be resistant to both INH and RIF than previously treated patients. Our findings could be helpful in the development of rapid molecular diagnostic methods and may improve our understanding of drug resistance in Hangzhou, aiding the development of precision medicine for TB and the disturbance of drug-resistant TB transmission.

## Figures and Tables

**Figure 1:**
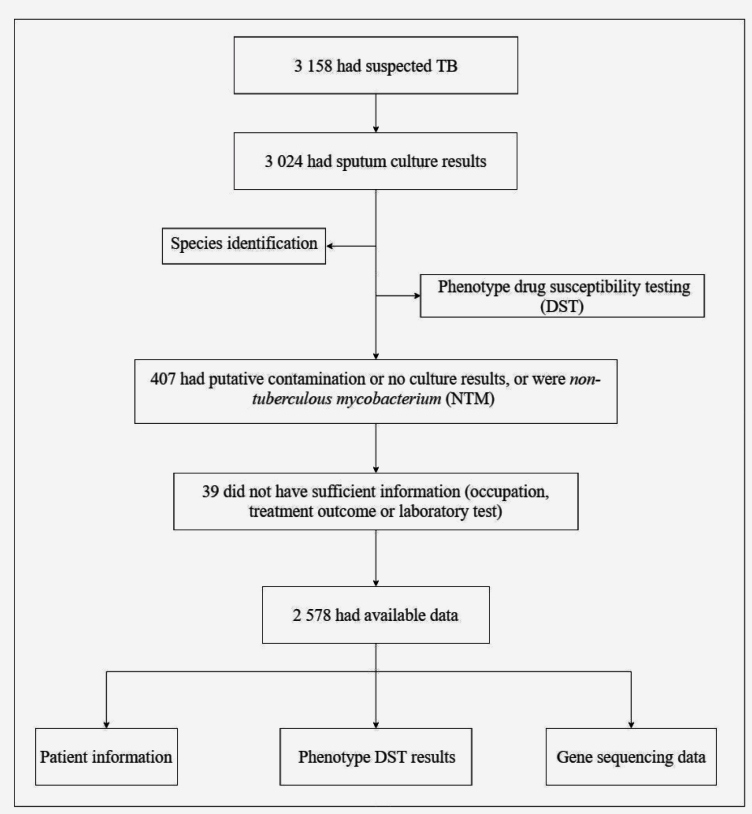
Supplemental file.
