# Erratum

**DOI:** 10.1590/0037-8682-0728B-2020

**Published:** 2022-10-21

**Revista da Sociedade Brasileira de Medicina Tropical/Journal of the Brazilian Society of Tropical Medicine**

**Title:** Drug-Resistant Mycobacterium tuberculosis Isolates from New and Previously Treated TB Patients in China, 2017-2019 

**54: (e0728-2021) 2021 - Page: 1/3 - doi:** 10.1590/0037-8682-0728-2020 

**CORRECT INDEXING OF AUTHORS:**

 Zeng Mei Chun^1^ and Jia Qing Jun^2^

Should read:

 Mei-Chun Zeng^1^ and Qing-Jun Jia^2^

**CONTACT AUTHOR**

**Corresponding author:** Zeng Mei Chun.

Should read:

**Corresponding author:** Mei-Chun Zeng.

